# The use of haemoglobin concentrations to assess physiological condition in birds: a review

**DOI:** 10.1093/conphys/cov007

**Published:** 2015-03-11

**Authors:** Piotr Minias

**Affiliations:** Department of Teacher Training and Biodiversity Studies, University of Łódź, Banacha 1/3, Łódź 90–237, Poland

**Keywords:** Bird, condition, fitness, haematology, haemoglobin concentration, parasite

## Abstract

Total blood haemoglobin concentration is increasingly being used to assess physiological condition in wild birds, although it has not been explicitly recognized how reliably this parameter reflects different components of individual quality. Thus, I reviewed over 120 published studies linking variation in haemoglobin concentrations to different measures of condition and other phenotypic or ecological traits. In most of the studied avian species, haemoglobin concentrations were positively correlated with other commonly used indices of condition, such as body mass and fat loads, as well as with quality of the diet. Also, chick haemoglobin concentrations reliably reflected the intensity of nest infestation by parasitic arthropods, and haemoglobin was suggested to reflect parasitism by haematophagous ectoparasites much more precisely than haematocrit. There was also some evidence for the negative effect of helminths on haemoglobin levels in adult birds. Finally, haemoglobin concentrations were found to correlate with such fitness-related traits as timing of arrival at breeding grounds, timing of breeding, egg size, developmental stability and habitat quality, although these relationships were not always consistent between species. In consequence, I recommend the total blood haemoglobin concentration as a relatively robust indicator of physiological condition in birds, although this parameter is also strongly affected by age, season and the process of moult. Thus, researchers are advised to control fully for these confounding effects while using haemoglobin concentrations as a proxy of physiological condition in both experimental and field studies on birds.

## Teaser

Total blood hemoglobin concentration is increasingly often used to assess physiological condition in wild birds, although its robustness has not been explicitly evaluated over a wide range of avian taxa. This review summarizes how reliable is hemoglobin concentration in assessing physiological condition and how it is related to various components of fitness and individual quality in birds.

## Introduction

Although animal physiologists have long relied on different blood markers to assess animal–environment interactions, the measurement of physiological parameters of blood is now increasingly being adopted to explain ecological and evolutionary processes occurring within vertebrate populations. The wide application of physiological approaches in basic ecology provided a tool that became of key importance in solving conservation problems, which laid the foundation for a new discipline called conservation physiology (Cooke and O'Connor, 2010; Cooke *et al.*, 2013). One of the most common applications of physiological methods in ecological studies and wildlife surveillance is to assess different components of individual condition and health (Stoot *et al.*, 2014). While many blood physiological parameters have been proposed to serve as proxies of condition in birds (e.g. plasma concentrations of different metabolites, reviewed by Brown, 1996), there is little consensus on which markers are sufficiently robust to reflect varying physiological condition reliably across different avian taxa. Among others, total blood haemoglobin concentration (usually expressed as the mass of haemoglobin per unit of blood) was suggested to be a condition-dependent trait that may be related to fitness in birds (e.g. O'Brien *et al.*, 2001; Bańbura *et al.*, 2007), but the generality of this pattern has not been evaluated explicitly.

Haemoglobin is encountered in all five kingdoms of organisms, ranging from single-chain globins in bacteria, algae, protozoa and plants to large, multisubunit and multidomain molecules in some invertebrates (Weber and Vinogradov, 2001). In the animal kingdom, haemoglobin occurs widely but sporadically in non-vertebrate species, being found in some 33% of presently known animal classes. In vertebrates, total blood haemoglobin concentration is considered to be the most important determinant of oxygen-carrying capacity. Although in mammals the amount of oxygen supplied to the tissues per unit time (measured by the amount of haemoglobin per total surface area of erythrocytes) is of greater physiological importance than the oxygen-carrying capacity of the blood (Kostelecka-Myrcha, 2002), the opposite situation is found in birds. The amount of haemoglobin per total surface area of erythrocytes remains constant among all avian species, even in differing physiological and environmental situations, as the changes in the number and size of erythrocytes always proceed in proportion to the changes in haemoglobin concentration. Such an adaptation seems to be optimal for the process of blood oxygen saturation in the lungs of birds, which is maintained at a relatively constant rate via the presence of air sacs and the cross-current exchange of gas between the air and the blood in the pulmonary capillaries (Schmidt-Nielsen, 1975; Kostelecka-Myrcha, 1997). Taking all this into account, the total blood haemoglobin concentration most appropriately reflects the potential of an avian organism to satisfy its oxygen requirements.

In consequence, high concentrations of haemoglobin improve aerobic capacity, whereas low concentrations are associated with the poor states of regenerative or non-regenerative anaemias. In birds, regenerative anaemias are usually caused by haemorrhage and haemolysis, most commonly attributed to parasitism (Boyd, 1951), injuries (Jaensch and Clark, 2004) and intoxication (Leighton *et al.*, 1983). In this kind of anaemia, the reductions in blood oxygen-carrying capacity typically result in increased erythropoiesis, resulting in the rapid production of large quantities of immature red blood cells (reticulocytes). In contrast, non-regenerative anaemias (with no response of bone marrow to the increased need for erythrocytes) are caused by chronic diseases, but also from nutritional deficiencies or starvation (Harrison and Harrison, 1986). For these reasons, haemoglobin concentration has been proposed as a robust indicator of physiological condition in birds (e.g. Bańbura *et al.*, 2007) and is increasingly being used to assess individual quality (*sensu*Wilson and Nussey, 2010) in this group of vertebrates (Pryke *et al.*, 2011; Crossin *et al.*, 2013; Minias *et al.*, 2015b).

The physiological condition of birds has also been commonly assessed using haematocrit (packed cell volume), which is the percentage of blood volume occupied by erythrocytes (Harrison and Harrison, 1986). However, reliability of haematocrit as a general measure of condition has recently been questioned. It has been acknowledged that haematocrit may not accurately reflect the concentration of erythrocytes in the blood, because it depends largely on the size of erythrocytes, and thus, it may not represent well the physiological condition of birds and other vertebrates (Fair *et al.*, 2007). In fact, haematocrit was shown not to be condition dependent in some of the studied avian species (Dawson and Bortolotti, 1997a; Hanssen *et al.*, 2003; Dzialowski and Sotherland, 2004). Consequently, alternative haematological parameters, including total blood haemoglobin concentration, have been proposed as potentially more reliable indicators of physiological condition and health in birds (O'Brien *et al.*, 2001). Although haematocrit and haemoglobin concentrations are expected to be correlated in birds (Velguth *et al.*, 2010), the strength of this correlation may be weak, because both parameters show different sensitivity to ecological factors (Kaliński, *et al.* 2011). In fact, haematocrit and haemoglobin concentration are based on different biological principles (blood cytology and blood biochemistry, respectively), and consequently, they may be indicative of different environmental and physiological processes (Bańbura *et al.*, 2007).

The aim of this review was to evaluate whether total blood haemoglobin concentration may be used reliably as an indicator of physiological condition in birds. The second objective of the study was to identify phenotypic and ecological factors that could affect haemoglobin concentrations, potentially reducing its applicability as a proxy of individual quality. For these purposes, I reviewed over 120 published studies linking variation in haemoglobin concentrations to age, sex, season, moult, nutritional state/condition, parasitism and other fitness-related traits.

## Age-related variation

Data on the changes in haemoglobin concentration throughout the post-hatching development of chicks were collected for 20 avian species (Supplementary material Table S1), mainly passerines (10 species) and seabirds (five species). All the studies that covered the entire period of chick development showed increases in haemoglobin concentration from hatching to fledging. Such patterns result from the process of haematopoiesis (production of blood cellular components) taking place in the liver, spleen and bone marrow, which starts at the embryonic stage of development and continues after hatching (Campbell, 1994). There was, however, considerable variation in developmental patterns of blood oxygen-carrying capacity between species (Simmons and Lill, 2006). In most altricial species, haemoglobin concentration tends to increase in a linear manner throughout nestling development (Kostelecka-Myrcha *et al.*, 1970, 1971, 1973; Bolton *et al.*, 1999). In some other altricial species (e.g. in Hirundininae) and some precocial species, haemoglobin concentration changes in a non-linear manner, because it increases rapidly during first days after hatching and thereafter the rate of change gradually slows down (Kostelecka-Myrcha and Jaroszewicz, 1993; Simmons and Lill, 2006). In these species, peak values of haemoglobin concentrations may be attained well before fledging and, afterwards, they decrease slightly to reach the adult level (Simmons and Lill, 2006). In contrast, the haemoglobin concentration in Procellariiformes shows an initial decrease, which is followed by relative stability during the most intensive growth period, and it starts to increase rapidly during mass recession, i.e. the period of body mass loss before fledging (Arnold *et al.*, 1999; O'Dwyer *et al.*, 2007). Similar patterns were also recorded in other seabirds, e.g. a slight decrease in haemoglobin concentration was found in Arctic tern (*Sterna paradisea*) chicks during the first days after hatching (Bech and Klaassen, 1996).

It seems that in many species the haemoglobin concentration attained at the time of fledgling increases gradually until adulthood. In most of the analysed species (72.2%), adult individuals were reported to have a significantly higher haemoglobin concentration in comparison to juveniles. Only one study on captive Masai ostriches (*Struthio camelus*) investigated changes in haemoglobin concentration throughout the entire first year of life (Samour *et al.*, 2011). The authors demonstrated that the haemoglobin concentration initially decreased slightly, but from the third month of life it started to increase at a constant rate until the age of at least 1 year, with the total increase being ∼40% (Samour *et al.*, 2011). Such patterns of changes may be characteristic for long-lived species with a long period of development. In fact, the only two studies on short-lived passerines found no differences in haemoglobin concentration between juveniles and adults (Puerta *et al.*, 1995; Archawaranon, 2005). There is little empirical data on the changes in haemoglobin concentration during the period of adulthood in birds. While three of four studied species showed no clear patterns of changes in haemoglobin concentrations throughout adulthood (Table 1), the study on feral pigeons (*Columba livia* f. *domestica*) demonstrated a significant decrease in haemoglobin concentration after the ninth year of age (Prinzinger and Misovic, 2010). In conclusion, more data are needed to establish the patterns of haemoglobin changes with age.

**Table 1:** Seasonal changes of haemoglobin concentrations in birds

Common name	Specific name	Order	Haemoglobin concentration	Reference
Zebra finch	Taeniopygia guttata*	PAS	Decreased during egg laying (F)	Wagner *et al.* (2008b)
Zebra finch	T. guttata*	PAS	Decreased during egg laying (F)	Willie *et al.* (2010)
Crimson finch	*Neochmia phaeton*	PAS	Decreased during egg laying and incubation (F)	Milenkaya *et al.* (2013)
Cory's shearwater	*Calonectris diomedea*	PRO	Increased from pre-laying to chick-rearing period (F)	Navarro *et al.* (2007)
Short-tailed shearwater	*Puffinus tenuirostris*	PRO	Increased from pre-laying to chick-rearing period	Davey *et al.* (2000)
Feral pigeon	*Columba livia* 'f. *domestica*	COL	Increased during egg laying	Kasprzak *et al.* (2006)
House sparrow	*Passer domesticus*	PAS	Increased between June and September (adults)	Puerta *et al.* (1995)
Noisy miner	*Manorina melanocephala*	PAS	Increased between breeding season and winter	Powell *et al.* (2013)
Four passerine species^a^		PAS	No changes between breeding season and winter	Breuer *et al.* (1995)
Superb fairywren	*Malurus cyaneus*	PAS	Decreased from breeding season to winter	Box *et al.* (2002)
Black-necked swan	*Cygnus melanocoryphus*	ANS	Decreased from August to February	Artacho *et al.* (2007)
Feral pigeon	*C. livia* f. *domestica*	COL	Increased over winter	Pavlak *et al.* (2005)
Mallard	Anas platyrhynchos*	ANS	Highest during winter and pre-laying period	Shave and Howard (1976)
Giant Canada goose	Branta canadensis maxima*	ANS	Highest during winter and pre-laying period	Shave and Howard (1976)
Common snipe	*Gallinago gallinago*	CHA	Increased over autumn migratory period	Minias *et al.* (2014)
Bar-tailed godwit	*Limosa lapponica*	CHA	Increased over spring migratory period	Piersma *et al.* (1996)

Abbreviations: ANS, Anseriformes; CHA, Charadriiformes; COL, Columbiformes; PAS, Passeriformes; PRO, Procellariiformes. Studies conducted in captivity are marked with an asterisk. Results valid for females only are marked with an ‘F’. ^a^Brown thornbill (*Acanthiza pusilla*), red-browed firetail (*Emblema temporalis*), eastern yellow robin (*Eopsaltria australis*) and superb fairywren (*Malurus cyaneus*).

## Sex-related variation

Data on differences in haemoglobin concentration between sexes in adult birds were available for 36 species (Supplementary material Table S2), mainly Charadriiformes (nine species) and Passeriformes (six species). In the large majority of species (86.1%), there was no significant difference between the sexes in the haemoglobin concentration of adult individuals, and this conclusion is consistent with the general patterns recorded for haematocrit (Fair *et al.*, 2007). In species where between-sex differences in haemoglobin concentration were found, females tended to have higher levels of haemoglobin, as found in two passerine (Kaliński *et al.*, 2012) and one procellariiform species (Myrcha and Kostelecka-Myrcha, 1980). A higher haemoglobin concentration in males compared with females was found in only one study on feral pigeons (Gayathri and Hegde, 1994), but two other studies on this species did not confirm the observation (Pavlak *et al.*, 2005; Kasprzak *et al.*, 2006).

Thus, it seems that any observed differences in haemoglobin concentrations between sexes may result primarily from temporal physiological processes associated with reproduction or moulting. In fact, a study on the crimson finch (*Neochmia phaeton*) indicated that such differences may appear only during specific reproductive stages, such as egg laying or incubation, although in general males and females have similar haemoglobin concentrations (Milenkaya *et al.*, 2013). Between-sex differences in haemoglobin concentration were found relatively often in nestlings, but it seems likely that these patterns could result largely from the different ages of male and female chicks sampled for the analyses.

## Seasonal changes

Decreasing haemoglobin concentrations during egg laying were recorded in females of at least two bird species (Table 1). Similar symptoms of anaemia during egg production, indicated by reduced haematocrit, have been documented in a wider range of avian taxa (DeGraw *et al.*, 1979; Jones, 1983; Gayathri and Hegde, 2006). The exact mechanisms responsible for these changes have not been explicitly recognized, and a few non-exclusive explanations have been proposed (Williams, 2004). A reduction in haemoglobin concentration during egg laying was hypothesized to result from transient suppression of erythropoiesis, so that energy for red blood cell production could be redirected to meet the elevated metabolic demands of the reproductive organs (Jones, 1983) and because components normally allocated to erythropoiesis could be required for egg production (Gayathri and Hegde, 2006; Kasprzak *et al.*, 2006). Decreased haemoglobin concentration during egg laying may also be caused by the production of yolk precursors and concurrent osmoregulatory adjustments (Salvante and Williams, 2002), which trigger an increase in plasma volume (haemodilution) due to osmotic movement of water from extracellular spaces into the blood (Reynolds and Waldron, 1999). In such circumstances, the haematocrit and blood haemoglobin concentration would be decreased, although the number of red blood cells would remain constant. Finally, anaemia during egg production may develop as a consequence of elevated levels of oestrogens (Williams *et al.*, 2005; Wagner *et al.*, 2008a), which suppress haematopoiesis by inhibiting proliferation and survival of blood cell precursors in bone marrow (Clermont and Schrar, 1979; Blobel and Orkin, 1996). In spite of these predictions, one of the studies on female feral pigeons showed an increased haemoglobin concentration during the period of egg laying, but this pattern was attributed to dehydration (Kasprzak *et al.*, 2006). Likewise, there was no pronounced decrease in haemoglobin concentrations of two procellariiform species during the egg-laying phase when compared with the pre-laying period (Davey *et al.*, 2000; Navarro *et al.*, 2007). In these species, levels of haemoglobin increased at a constant rate from the pre-laying to the chick-rearing period, which was attributed to the high costs of the very long foraging trips undertaken by parents when feeding nestlings and the preparation for migration that is undertaken soon after the conclusion of reproductive activities (Davey *et al.*, 2000).

Migratory behaviour was, indeed, found to have a strong effect on haemoglobin concentration in birds. For example, haemoglobin concentrations increased considerably over the migratory period in two studied wader species, which was associated with increasing fat loads necessary for long-distance flights (Piersma *et al.*, 1996; Minias *et al.*, 2014). In other avian taxa, a requirement for improved oxygen-carrying capacity during migration was suggested by elevated haematocrit levels (Bairlein and Totzke, 1992; Prats *et al.*, 1996). In contrast, changes in haemoglobin concentration during the autumn period proceeded in a different manner in non-migratory species. In the superb fairywren (*Malurus cyaneus*), haemoglobin concentration was found to decrease from summer to winter (Box *et al.*, 2002), although the other study on this species found no changes over autumn (Breuer *et al.*, 1995). In other non-migratory passerine species, there were no changes in haemoglobin levels between seasons (Breuer *et al.*, 1995). There is also little empirical support for increased haemoglobin concentrations during winter. It has been hypothesized that birds should have the highest oxygen-carrying capacity of blood during winter period, because oxygen uptake should be elevated during increased thermogenesis (Swanson, 1990). The only evidence for such relationships comes from free-living feral pigeons (Pavlak *et al.*, 2005) and noisy miners (*Manorina melanocephala*; Powell *et al.*, 2013), as well as from captive waterfowl (Shave and Howard, 1976).

## Moult

Data on the changes of haemoglobin concentration in relationship to moult were collected for 11 bird species, including only two species of passerines (Table 2). The general pattern emerging from these studies indicates that the haemoglobin concentration in blood decreases at the beginning of moult, but it tend to increase as the moult progresses to reach pre-moult levels after the completion of feather replacement. Given that moult is associated with extensive vascularization of growing quills, plasma volume must increase substantially during this period. Thus, the decreased haemoglobin concentration during moult initiation may be explained by increasing plasma volume that is not accompanied by a compensatory increase in the total number of erythrocytes (Chilgren and DeGraw, 1977). Erythropoiesis is unlikely to be suppressed though, because there is no evidence for a reduction in total the number of erythrocytes during the period of feather replacement in birds (Chilgren and DeGraw, 1977; DeGraw and Kern, 1985).

**Table 2:** Changes of haemoglobin concentrations in response to moult in birds

Common name	Specific name	Order	Moult	Haemoglobin concentration	Reference
Common snipe	*G. gallinago*	CHA	Post-juvenile	Low at the beginning of moult, increased as moult progressed	Minias *et al.* (2014)
Emperor penguin	Aptenodytes forsteri*	SPH	Post-juvenile	Higher in post-moult period than in pre-moult period	Ponganis *et al.* (1999)
Bar-tailed godwit	*L. lapponica*	CHA	Pre-breeding	Higher during moult	Piersma *et al.* (1996)
Dunlin	*Calidris alpina*	CHA	Pre-breeding	Decreased as moult progressed	Verhulst *et al.* (2002)
Mallard	*A. plathyrynchos*	ANS	Post-breeding	Lower during moult	Driver (1981)
Feral pigeon	*C. livia* f. *domestica*	COL	Post-breeding	Lower during moult	Kasprzak *et al.* (2006)
Pale-breasted thrush	*Turdus leucomelas*	PAS	Post-breeding	Lower during moult	Lobato *et al.* (2011)
Little penguin	*Eudyptula minor*	SPH	Post-breeding	Lower during moult, increased after moult	Mortimer and Lill (2007)
Rockhopper Penguin	*Eudyptes chrysocome*	SPH	Post-breeding	No effect of moult, decreased after moult	Hawkey *et al.* (1989)
Common starling	*Sturnus vulgaris*	PAS	Post-breeding	No effect of moult	Prinzinger and Hakimi (1997)
Common snipe	*G. gallinago*	CHA	Post-breeding	Low at the beginning of moult, increased as moult progressed	Minias *et al.* (2014)
Magellanic penguin	*Spheniscus magellanicus*	SPH	Post-breeding	Lower in post-moult period than in pre-moult period	Hawkey *et al.* (1989)

Abbreviations: ANS, Anseriformes; CHA, Charadriiformes; COL, Columbiformes; PAS, Passeriformes; SPH, Spenisciformes. Studies conducted in captivity are marked with an asterisk.

Decreased haemoglobin levels at the beginning of moult were recorded for young (partial post-juvenile moult) and adult (complete post-breeding moult) common snipe (*Gallinago gallinago*; Minias *et al.*, 2014). However, post-breeding moult in this species was associated with much greater change in haemoglobin concentration. Adult snipe had 12% higher haemoglobin concentrations after moult compared with birds at the beginning of moult. In contrast, the haemoglobin concentration of juvenile snipe decreased by only 2% at the beginning of post-juvenile moult (Minias *et al.*, 2014).

Despite the fact that most of the studies indicated lower haemoglobin concentrations during moult, some exceptions to this general pattern were also observed. In two species, no moult-related changes in haemoglobin concentration were recorded (Hawkey *et al.*, 1989; Prinzinger and Hakimi, 1997). In bar-tailed godwits (*Limosa lapponica*), individuals moulting breast feathers at a spring stopover site had a higher haemoglobin concentration in comparison to non-moulting individuals; however, early moult of body feathers was associated with high individual quality, so this relationship could be purely correlative in nature (Piersma *et al.*, 1996).

## Nutritional state and condition

There is fairly sound empirical evidence that haemoglobin concentration may be considered a reliable proxy of condition (defined in a broad sense as a composite of factors including nutritional state, level of health and physiological performance) in free-living birds. In 12 avian species, positive correlations were found between haemoglobin concentration and size-corrected or uncorrected body mass and fat loads, while no relationship has been reported for only one species (Table 3). Fat loads were found to be a good predictor of haemoglobin concentrations in migratory waders at both intra- and interspecific levels (Minias *et al.*, 2013a). Given that migrants have to accumulate large fat reserves before departure on long-distance flights, elevated haemoglobin concentrations may be necessary to meet the high metabolic costs of travelling with a large fat burden. Thus, the relationship between haemoglobin concentration and body mass or fat loads may not be purely correlative but may reflect physiological adjustments. A positive relationship between body mass and haemoglobin concentrations may also result from suppressed erythropoiesis during starvation (Campbell, 1994).

**Table 3:** Relationships of haemoglobin concentrations with different measures of nutritional state and condition in birds

Common name	Specific name	Order	Age	Parameter	Haemoglobin concentration	Reference
Welcome swallow	*Hirundo neoxena*	PAS	Ad.	Body mass	+	Lill *et al.* (2013b)
Bar-tailed godwit	*L. lapponica*	CHA	Ad.	Body mass	+	Piersma *et al.* (1996)
Great tit	*Parus major*	PAS	Ad.	Body mass	+	Dufva (1996)
Grey-headed albatross	*Thalassarche chrysostoma*	PRO	Ad.	Body mass	+	Crossin *et al.* (2013)
Pale-breasted thrush	*Turdus leucomelas*	PAS	Ad.	Body mass	+	Lobato *et al.* (2011)
Welcome swallow	*H. neoxena*	PAS	Pull.	Size-corrected body mass	+	Lill *et al.* (2013a)
Gould's petrel	*Pterodroma leucoptera*	PRO	Pull.	Size-corrected body mass	+	O'Dwyer *et al.* (2007)
Bar-tailed godwit	*L. lapponica*	CHA	Ad.	Size-corrected body mass	+	Landys-Ciannelli *et al.* (2002)
Crimson finch	*N. phaeton*	PAS	Ad.	Size-corrected body mass/fat load	(−)	Milenkaya *et al.* (2013)
Six wader species^a^		CHA	Juv.	Size-corrected body mass/fat load	+	Minias *et al.* (2013a)
Common snipe	*G. gallinago*	CHA	Juv./Ad.	Plasma concentrations of metabolites	+ TP, (−) GL	Minias *et al.* (2014)
Cory's shearwater	*C. diomedea*	PRO	Ad.	Plasma concentrations of metabolites	(−) TG, CHOL, TP, UA, UR	Navarro *et al.* (2007)
Crimson finch	*N. phaeton*	PAS	Ad.	Plasma concentrations of metabolites	(−) TP	Milenkaya *et al.* (2013)
Whiskered tern	*Chlidonias hybrida*	CHA	Ad.	Plasma concentrations of metabolites	− GL	Minias (2014)
Parrot finch	Erythrura trichroa*	PAS	Pull.	Diet quality	+	Pryke and Rollins (2012)
Gouldian finch	Erythrura gouldiae*	PAS	Pull.	Diet quality	+	Pryke *et al.* (2011)
Gouldian finch	E. gouldiae*	PAS	Ad.	Diet quality	+	Pryke *et al.* (2012)
Zebra finch	T. guttata*	PAS	Ad.	Diet quality	(−)	Wagner *et al.* (2008b)
Great tit	*P. major*	PAS	Pull.	Food supplementation	(−)	Bańbura *et al.* (2011)
Adélie penguin	*Pygoscelis adeliae*	SPH	Ad.	Fast duration	+	Vleck and Vleck (2002)

Abbreviations: Ad., adult; CHA, Charadriiformes; CHOL, cholesterol; GL, glucose; Juv., juvenile; PAS, Passeriformes; PRO, Procellariiformes; Pull., pullus; SPH, Sphenisciformes; TG, triglycerides; TP, total protein; UA, uric acid; UR, urea. Studies conducted in captivity are marked with an asterisk. Note: ‘+’ indicates a positive relationship; ‘−’ indicates a negative relationship; and ‘(−)’ indicates no relationship. ^a^Common sandpiper (*Actitis hypoleucos*), dunlin (*Calidris alpina*), common snipe (*Gallinago gallinago*), ruff (*Philomachus pugnax*), spotted redshank (*Tringa erythropus*) and green sandpiper (*Tringa ochropus*).

Strong support for the reliability of haemoglobin concentrations as a predictor of condition comes from experimental studies on captive passerines, where haemoglobin concentration was positively affected by the quality of diet in both nestlings (Pryke *et al.*, 2011; Pryke and Rollins, 2012) and adults (Pryke *et al.*, 2012). In contrast, the only one study on captive zebra finches (*Taeniopygia guttata*) showed no effect of diet on haemoglobin concentration (Wagner *et al.*, 2008b). No effect of food supplementation on haemoglobin concentration was also found for a free-living population of great tits (*Parus major*), although it was probably due to non-restrictive abundance of natural food resources (Bańbura *et al.*, 2011). It must be acknowledged, however, that haemoglobin concentration may not provide a good indication of body condition during serious dehydration of an organism, as was shown for fasting Adélie penguins (*Pygoscelis adeliae*; Vleck and Vleck, 2002).

Much less consistent are relationships between the concentrations of haemoglobin and different plasma metabolites. The only reported positive correlations were found for the common snipe, where haemoglobin concentration was correlated with the plasma concentration of total proteins (Minias *et al.*, 2014). No significant relationships were found in other species (Navarro *et al.*, 2007; Milenkaya *et al.*, 2013), and in the whiskered tern (*Chlidonias hybrida*) a negative correlation between haemoglobin concentration and plasma glucose level was recorded (Minias, 2014); this pattern was attributed to the oxidative stress associated with hyperglycaemia, which may reduce synthesis of erythropoietin, a hormone responsible for red blood cell production (Winkler, *et al.* 1999).

## Parasitism

Data on the effects of haematophagous nest ectoparasites on haemoglobin concentrations in nestlings are fully consistent among studied taxa and unequivocally indicate negative effects of parasitism on the haematological condition of chicks. In total, all but one reviewed study on passerines (*n* = 12) demonstrated negative relationships between chick haemoglobin concentration and the intensity of nest infestation by parasitic arthropods, such as *Protocalliphora* and *Philornis* fly larvae or mites (Table 4). There was only one study that found no reduction in haemoglobin concentration in response to infestation by the mite *Ornithonyssus bursa* (Arachnida) on the common starling (*Sturnus vulgaris*; Powlesland, 1977); however, a more recent study on this species demonstrated a negative effect of infestation by *Ornithonyssus sylviarum* on chick haemoglobin levels (Clark and Mason, 1988).

**Table 4:** Changes of haemoglobin concentrations in response to parasite pressure in birds

Common name	Specific name	Order	Age	Parasite	Haemoglobin concentration	Reference
Magpie	*Pica pica*	PAS	Pull.	Blow fly larvae (*Protocalliphora asiovora*)	−	Whitworth and Bennett (1992)
Pied flycatcher	*Ficedula hypoleuca*	PAS	Pull.	Blow fly larvae (*Protocalliphora azurea*)	−	Belskii *et al.* (2005)
Bank swallow	*Riparia riparia*	PAS	Pull.	Blow fly larvae (*Protocalliphora chrysorrhoea*)	−	Whitworth and Bennett (1992)
House wren	*Troglodytes aedon*	PAS	Pull.	Blow fly larvae (*Protocalliphora parorum*)	−	O'Brien *et al.* (2001)
Eastern bluebird	*Sialis sialis*	PAS	Pull.	Blow fly larvae (*Protocalliphora* sp.)	−	Hannam (2006)
Medium ground finch	*Geospiza fortis*	PAS	Pull.	Fly larvae (*Philornis downsi*)	−	Fessl *et al.* (2006)
Small ground finch	*Geospiza fuliginosa*	PAS	Pull.	Fly larvae (*P. downsi*)	−	Fessl *et al.* (2006)
Small ground finch	*G. fuliginosa*	PAS	Pull.	Fly larvae (*P. downsi*)	−	Dudaniec *et al.* (2006)
Great tit	*P. major*	PAS	Pull.	Parasitic arthropods (fleas, blow flies and mite)	−	Słomczyński *et al.* (2006)
Eastern bluebird	*Sialia sialis*	PAS	Pull.	Mite (*Dermanyssus prognephilus*)	−	Carleton (2008)
Common starling	*S. vulgaris*	PAS	Pull.	Mite (*Ornithonyssus sylviarum*)	−	Clark and Mason (1988)
Common starling	*S. vulgaris*	PAS	Pull.	Mite (*Ornithonyssus bursa*)	(−)	Powlesland (1977)
Herring gull	Larus argentatus*	CHA	Pull.	Tapeworm (*Diphyllobothrium dendriticum*)	−	Mazur *et al.* (2007)
Skylark	*Alauda arvensis*	PAS	Ad.	Gastrointestinal helminths (*Heterakis gallinae* and *Ascaridia galli*)	−	Khan *et al.* (2006)
Robin	*Erithacus rubecula*	PAS	Ad.	Ticks	−	Norte *et al.* (2013)
Little penguin	*Eudyptula minor*	SPH	Ad.	*Babesia*	−	Sergent *et al.* (2004)
Superb fairywren	*M. cyaneus*	PAS	Ad.	*Haemoproteus*	(−)	Colombelli-Négrel and Kleindorfer (2008)
Black-fronted piping-guan	Aburria jacutinga*	GAL	Ad.	*Haemoproteus*, *Plasmodium*	(−)	Motta *et al.* (2013)
107 passerine species		PAS	Ad.	*Haemoproteus*, *Plasmodium*, *Leucocytozoon*, *Trypanosoma*, microfilariae	(−)	Booth and Elliott (2002)
Great tit	*P. major*	PAS	Ad.	*Haemoproteus*, *Plasmodium*, *Leucocytozoon*, *Trypanosoma*, *Hepatozoon*	(−)	Dufva (1996)
Great tit	*P. major*	PAS	Pull.	*Haemoproteus*, *Plasmodium*, *Leucocytozoon*	−	Krams *et al.* (2013)
Feral pigeon	*C. livia* f. domestica*	COL	Pull.	*Trypanosoma evansi*	−	Mandal *et al.* (2008)

Abbreviations: Ad., adult; CHA, Charadriiformes; COL, Columbiformes; GAL, Galliformes; Juv., juvenile; PAS, Passeriformes; Pull., pullus; SPH, Sphenisciformes. Studies conducted in captivity are marked with an asterisk. Note: ‘−’ indicates a decrease in haemoglobin concentration; and ‘(−)’ indicates no change in haemoglobin concentration.

In contrast, the response of nestling haematocrit to the parasitism by nest arthropods follows no clear pattern (reviewed by Fair *et al.*, 2007). Although some of the studies indicated a negative impact of parasitic burden on chick haematocrit (Moreno *et al.*, 1999; Potti *et al.*, 1999; Simon *et al.*, 2004), a relatively large number of the studied species did not show any significant relationships. It has been reported that parasitism by *Carnus haemapterus* (Diptera: Carnidae) had no effect on haematocrit of American kestrel (*Falco sparverius*) chicks (Dawson and Bortolotti, 1997b), the presence of the hen flea (*Ceratophyllus gallinae*; Ceratophyllidae) did not reduce haematocrit in blue tits (*Cyanistes caeruleus*; Tripet and Richner, 1999), and experimental infestation of nestling barn swallows (*Hirundo rustica*) with hippoboscid louse flies (*Ornithomyia biloba*; Diptera: Hippoboscidae) also had no impact on this parameter (Saino *et al.*, 1998). Taking all these findings into account, it may be concluded that haemoglobin concentration seems to reflect parasitism by haematophagous ectoparasites much more precisely than haematocrit. This hypothesis was first put forward by O'Brien *et al.* (2001), who demonstrated that house wren (*Troglodytes aedon*) nestlings heavily parasitized by larvae of the blow fly (*Protocalliphora parorum*) showed no reduction in haematocrit, but a significant 28% reduction in haemoglobin level. This confirmed the earlier observations of Johnson and Albrecht (1993), who found that heavily parasitized house wren nestlings retained normal haematocrits. These observations suggested that haemoglobin concentration and haematocrit may respond in a different manner to blood loss. In fact, blood loss in birds is immediately followed by production of immature blood cells, reticulocytes, which rapidly complement haematocrit. However, the quantity of haemoglobin synthesized in reticulocytes does not exceed 20% of the level found in the mature red blood cells, so blood haemoglobin concentration is complemented with a substantial delay. The higher sensitivity of haemoglobin concentration to infestation by ectoparasites clearly indicates that it should be preferred over haematocrit in studies investigating the health consequences of parasitic pressure in birds.

Only two studies investigated the impact of helminths on blood haemoglobin concentration in birds, and both demonstrated reduced haemoglobin levels in response to parasitic infection (Khan *et al.*, 2006; Mazur *et al.*, 2007). Detrimental effects of intestinal nematodes *Ascaridia galli* on the haematological condition of birds were confirmed by reduced haematocrits in experimentally infected young male red jungle fowl (*Gallus gallus*; Johnsen and Zuk, 1998). In contrast, there is little support for the effects of haematozoan infections on blood haemoglobin concentrations in birds (Table 4). A robust study by Booth and Elliott (2002) found no within-family and within-species relationships between infections by haematozoa and blood haemoglobin concentrations in 688 Neotropical passerines from over 100 species during spring migration. Likewise, no negative effect of haematozoa on haemoglobin concentrations was found in breeding great tits (Dufva, 1996) and superb fairywrens (Colombelli-Négrel and Kleindorfer, 2008). In contrast, reduced haemoglobin concentration was found in nestling great tits infected with haematozoa (Krams, *et al.* 2013), and a similar effect was found in captive feral pigeon nestlings infected with *Trypanosoma evansi* (Mandal *et al.*, 2008). Thus, the possibility cannot be excluded that haematozoan infections have more detrimental consequences for nestlings, when compared with adult birds, but more empirical studies are needed to confirm this hypothesis. There is much more information on the impact of haematozoa on haematocrit in free-living birds; nevertheless, the results are also inconsistent among the studied taxa (reviewed by Fair *et al.*, 2007). It also remains unclear whether anaemic states are caused directly by haematozoan infections or indirectly by the blood loss caused by their vectors.

## Other fitness-related traits

There is little and inconsistent information on whether haemoglobin concentration correlates with reproductive performance of adult birds. There is some evidence that individuals with higher haemoglobin concentrations return earlier from wintering quarters to breeding grounds (Crossin *et al.*, 2013) and initiate breeding earlier in the season (Minias, 2014); however, in some other species no such relationships were found (Crossin *et al.*, 2012). Likewise, breeding grey-headed albatrosses (*Thalassarche chrysostoma*) had higher haemoglobin concentrations in comparison to non-breeders (Crossin *et al.*, 2013), but there was no such association in the black-browed albatross (*Thalassarche melanophris*; Crossin *et al.*, 2012). It was also found that adult whiskered terns with higher haemoglobin concentrations had clutches with larger eggs (Minias, 2014). Surprisingly, there are no data on whether the physiological condition of adult birds as assessed by blood haemoglobin concentration is correlated with such basic reproductive parameters as clutch size, hatching success or breeding output.

There are similar inconsistencies in physiological data collected for nestlings. In most studied species, chick haemoglobin concentration did not vary with brood size (Table 5). In contrast, there was a significant negative correlation between these traits reported for the Montagu's harrier (*Circus pygargus*; Limiñana *et al.*, 2009) and a positive correlation reported for the common tern (*Sterna hirundo*; Minias *et al.*, 2015b). A negative relationship between chick haemoglobin concentration and brood size may be explained by increased competition for food in larger broods, resulting in reduced physiological condition of the offspring. Nevertheless, brood size is usually correlated with adult quality, because high-quality parents are capable of rearing more offspring per breeding attempt, due to better foraging efficiency and higher breeding experience. In such circumstances, we could expect that intensified within-brood competition for food may be well compensated by higher feeding capacities of adults, explaining the lack of significant relationships between brood size and chick haemoglobin concentrations or even leading to increased haemoglobin concentrations in larger broods. Finally, only one out of four studies on passerine species showed a significant positive correlation between the haemoglobin concentration of nestlings and their survival (Table 5). A seasonal decline in chick haemoglobin concentration was reported for only one species (Minias *et al.*, 2015b).

**Table 5:** Relationships of haemoglobin concentrations with different fitness-related traits in birds

Common name	Specific name	Order	Age	Parameter	Haemoglobin concentration	Reference
Grey-headed albatross	*T. chrysostoma*	PRO	Ad.	Timing of arrival at breeding grounds	−	Crossin *et al.* (2013)
Black-browed albatross	*Thalassarche melanophris*	PRO	Ad.	Timing of arrival at breeding grounds	(−)	Crossin *et al.* (2012)
Whiskered tern	*C. hybrida*	CHA	Ad.	Breeding date	−	Minias (2014)
Grey-headed albatross	*T. chrysostoma*	PRO	Ad.	Breeding status	Breeders > non-breeders	Crossin *et al.* (2013)
Back-browed albatross	*T. melanophris*	PRO	Ad.	Breeding status	(−)	Crossin *et al.* (2012)
Whiskered tern	*C. hybrida*	CHA	Ad.	Egg size	+	Minias (2014)
Common tern	*Sterna hirundo*	CHA	Pull.	Hatching date	−	Minias *et al.* (2015b)
White stork	*Ciconia ciconia*	CIC	Pull.	Brood size	(−)	Kaminski *et al.* (2014)
Blue tit	*Cyanistes caeruleus*	PAS	Pull.	Brood size	(−)	Bańbura *et al.* (2008)
Tree swallow	*Tachycineta biolor*	PAS	Pull.	Brood size	(−)	Burness *et al.* (2000)
Montagus's harrier	*Circus pygargus*	ACC	Pull.	Brood size	−	Limiñana *et al.* (2009)
Common tern	*S. hirundo*	CHA	Pull.	Brood size	+	Minias *et al.* (2015b)
Blue tit	*C. caeruleus*	PAS	Pull.	Survival	+	Bańbura *et al.* (2007)
Great tit	*P. major*	PAS	Pull.	Survival	(−)	Nadolski *et al.* (2006)
Great tit	*P. major*	PAS	Pull.	Survival	(−)	Eeva *et al.* (2000)
Pied flycatcher	*Ficedula hypoleuca*	PAS	Pull.	Survival	(−)	Eeva *et al.* (2000)
Blue tit	*C. caeruleus*	PAS	Pull.	Habitat	Parkland < woodland	Bańbura *et al.* (2007)
Great tit	*P. major*	PAS	Pull.	Habitat	Parkland < woodland	Kaliński *et al.* (2009)
Noisy miner	*M. melanocephala*	PAS	Ad.	Habitat	Urban < rural	Powell *et al.* (2013)
House sparrow	*Passer domesticus*	PAS	Ad.	Habitat	Urban < rural	Herrera-Dueñas *et al.* (2014)
13 passerine species^a^		PAS	Ad.	Habitat quality and fragmentation	(−)	Amos *et al.* (2013)
Black-necked swan	*C. melanocoryphus*	ANS	Ad.	Habitats of varying food availability	(−)	Norambuena and Bozinovic (2009)
Common tern	*S. hirundo*	CHA	Pull.	Social environment (colony size)	−	Minias *et al.* (2015b)
Gouldian finch	E. gouldiae*	PAS	Pull.	Social environment (level of aggression)	−	Pryke and Griffith (2009)
Common snipe	*G. gallinago*	CHA	Juv.	Developmental stability (moult symmetry)	+	Minias *et al.* (2013b)
Common snipe	*G. gallinago*	CHA	Ad.	Developmental stability (wing shape symmetry)	+	Minias *et al.* (2014)
Whiskered tern	*C. hybrida*	CHA	Ad.	Heterozygosity	(−)	Minias *et al.* (2015a)

Abbreviations: ACC, Accipitriformes; Ad., adult; ANS, Anseriformes; CHA, Charadriiformes; CIC, Ciconiiformes; Juv., juvenile; PAS, Passeriformes; PRO, Procellariiformes; Pull., pullus. Studies conducted in captivity are marked with an asterisk. Note: ‘+’ indicates a positive relationship; ‘−’ indicates a negative relationship; and ‘(−)’ indicates no relationship. ^a^Buff-rumped thornbill (*Acanthiza reguloides*), dusky woodswallow (*Artamus cyanopterus*), brown treecreeper (*Climacteris picumnus*), grey shrikethrush (*Colluricincla harmonica*), eastern yellow robin (*Eopsaltria australis*), fuscous honeyeater (*Lichenostomus fuscus*), yellow-tufted honeyeater (*Lichenostomus melanops*), white-plumed honeyeater (*Lichenostomus penicillatus*), superb fairywren (*Malurus cyaneus*), brown-headed honeyeater (*Melithreptus brevirostris*), spotted pardalote (*Pardalotus punctatus*), striated pardalote (*Pardalotus striatus*) and weebill (*Smicrornis brevirostris*).

Some empirical evidence has been presented for habitat-related differences in haemoglobin concentrations within passerine populations. Nestlings of two tit species were found to have higher haemoglobin concentrations in woodland in comparison to parkland, and these differences were suggested to reflect variation in food availability (Bańbura *et al.*, 2007; Kaliński *et al.*, 2009). In two other passerines, adults from rural populations had higher blood haemoglobin concentrations in comparison to urban birds (Herrera-Dueñas *et al.* 2014; Powell *et al.*, 2013). Given that haemoglobin is considered a reliable oxidative stress marker, these patterns were explained by differences in pollution between rural and urban areas. In contrast, no evidence for a relationship between haemoglobin concentrations and habitat fragmentation were found for 13 woodland passerines (Amos *et al.*, 2013).

Only two studies investigated the effects of different social environments on haemoglobin concentrations in birds. A cross-fostering experiment conducted on a colonial waterbird species demonstrated that offspring raised in larger nesting aggregations had lower haemoglobin concentrations, probably due to increased social stress, although the effects of increased parasitism could not be excluded (Minias *et al.*, 2015b). The second study, on Gouldian finches (*Erythrura gouldiae*), showed that offspring raised in experimentally manipulated highly competitive environments had lower haemoglobin concentrations in comparison to offspring whose parents were subject to reduced levels of competition and aggression (Pryke and Griffith, 2009).

Blood haemoglobin levels were also found to covary with developmental stability (defined as the ability of individuals to undergo stable development of their phenotype in a range of environmental conditions, following Møller, 1997), although the evidence for such a relationship came only from studies on common snipe. It was demonstrated in young snipe that a low haemoglobin concentration had a negative effect on post-juvenile moult symmetry (Minias *et al.*, 2013b). This relationship was speculated to be causal, because low oxygen-carrying capacity of the blood may cause organismal hypoxia during long migratory flights, leading to disruptions in developmental homeostasis (Minias *et al.*, 2013b). In contrast, in adult snipe the haemoglobin concentration was associated with symmetry in the wing shape, but this relationship was suggested purely to be correlative, indicating lower genetic and phenotypic quality of birds with lower levels of developmental stability (Minias *et al.*, 2014). Relationships between other measures of genetic quality, such as heterozygosity, and haemoglobin concentrations were investigated only in the whiskered tern, but no significant correlation has been reported (Minias *et al.*, 2015a).

## Conclusions

On the basis of the reviewed literature, it seems safe to conclude that the total blood haemoglobin concentration may be used as a relatively robust indicator of physiological condition in birds. In most of the studied taxa, the concentration of haemoglobin was correlated with other commonly used indices of condition, such as body mass (corrected and uncorrected for size) and fat loads. Haemoglobin concentrations were also found to increase with a high-quality diet, suggesting that they may directly reflect the nutritional state of individuals. Haemoglobin concentrations reliably reflected parasitic pressure from haematophagous ectoparasites and they correlated with a number of fitness-related traits, although these relationships were not always consistent between species. In spite of this, there are additional sources of variation in haemoglobin levels, which have to be taken into account while using this parameter as a proxy of physiological condition. Due to increasing erythropoiesis, haemoglobin concentrations were found to increase rapidly with age, especially at the nestling stage and possibly also during the post-fledging period. Although in general there was no difference in haemoglobin concentration between sexes, some intersexual variation may appear during specific reproductive stages, such as egg laying or incubation. In fact, anaemic states associated with egg production are primarily responsible for seasonal patterns in haemoglobin concentrations. In contrast, the requirement for improved oxygen-carrying capacity in migrating birds is responsible for increased haemoglobin concentrations often recorded during the spring and autumn periods. Haemoglobin concentrations were also found to be affected by the process of feather replacement, usually decreasing at the beginning of moult and then gradually increasing to the pre-moult level. In conclusion, factors such as age, season and moult are recommended to be controlled for fully, if haemoglobin concentrations are expected to reflect variation in the physiological condition of birds reliably. It remains to be established whether and how strongly condition assessed by haemoglobin concentration may affect direct fitness components, i.e. reproductive output and survival, in natural bird populations and whether it is related to the genetic quality of individuals.

## Supplementary material

Supplementary material is available at *Conservation Physiology* online.

## Supplementary Material

Supplementary Data
