# Supplementary material for: The use of haemoglobin concentrations to assess physiological condition in birds: a review
Source: Conserv Physiol. 2015 Mar 11;3(1):cov007. doi: 10.1093/conphys/cov007 (PMC4778452; doi:10.1093/conphys/cov007)
Supplement: Supplementary Data [file cov007supp.zip › cov007supp.pdf]

Electronic Supplementary Material

The use of hemoglobin concentrations to assess physiological condition in birds: a review

Piotr Minias

Department of Teacher Training and Biodiversity Studies, University of Łódź, Banacha 1/3, 90–237, Łódź, Poland. E-mail: pminias@op.pl

Table S1: Age-related differences in hemoglobin concentrations in birds. Studies conducted in captivity are marked with an asterisk. Note: "+" indicates an increase in Hb concentration, "-" indicates a decrease in Hb concentration, and "(-)" indicates no change in Hb concentration.

Common name	Specific name	Order[A]	Age	Hb concentration	Reference
Arctic Tern	*Sterna paradisea*	CHA	Pull. (0-9 days)	-	Bech and Klaassen 1996
Barnacle Goose	*Branta leucopsis**	ANS	Pull.	+	Deaton et a. 1998
Pigeon Guillemot	*Cepphus columba*	CHA	Pull.	+	Haggblom *et al.* 1988
White Stork	*Ciconia ciconia*	CIC	Pull.	+	Montesinos *et al.* 1997
Feral Pigeon	*Columba livia* f. *domestica*	COL	Pull.	+	Gayathri *et al.* 2004
House Martin	*Delichon urbica*	PAS	Pull.	+	Kostelecka-Myrcha and Jaroszewicz 1993
Welcome Swallow	*Hirundo neoxena*	PAS	Pull.	+	Simmons and Lill 2006
Noisy Miner	*Manorina melanocephala*	PAS	Pull.	+	Bolton *et al.* 1999
Rainbow Bee-eater	*Merops ornatus*	COR	Pull.	+	Eklom and Lill 2006a
Wilson's Storm Petrel	*Oceanites oceanicus*	PRO	Pull.	+	Kostelecka-Myrcha and Myrcha 1989
Great Tit	*Parus major*	PAS	Pull.	+	Kostelecka-Myrcha *et al.* 1973
House Sparrow	*Passer domesticus*	PAS	Pull.	+	Kostelecka-Myrcha *et al.* 1971
Tree Sparrow	*Passer montanus*	PAS	Pull.	+	Kostelecka-Myrcha *et al.* 1970
Tree Sparrow	*Passer montanus*	PAS	Pull.	+	Kostelecka-Myrcha *et al.* 1996
Fairy Martin	*Petrochelidon ariel*	PAS	Pull.	+	Simmons and Lill 2006
Magpie	*Pica pica*	PAS	Pull.	+	Whitworth and Bennett 1992
Gould's Petrel	*Pterodroma leucoptera*	PRO	Pull.	+	O'Dwyer *et al.* 2007
Short-tailed Shearwater	*Puffinus tenuirostris*	PRO	Pull.	+	Arnold *et al.* 1999
Bank Swallow	*Riparia riparia*	PAS	Pull.	+	Whitworth and Bennett 1992
Spotted Dove	*Streptopelia chinensis*	COL	Pull.	+	Eklom and Lill 2006b
Common Starling	*Sturnus vulgaris*	PAS	Pull.	+	Kostelecka-Myrcha *et al.* 1972
Common Pheasant	*Phasianus colchicus**	GAL	Pull. vs. Juv.	+	Keçeci and Çöl 2011
American Flamingo	*Phoenicopterus ruber**	PHO	Pull. vs. Juv.	+	Puerta *et al.* 1992
Lesser Rhea	*Pterocnemia pennata**	RHE	Pull. vs. Juv.	+	Reissig *et al.* 2002
Small Ground Finch	*Geospiza fuliginosa*	PAS	Pull. vs. Ad.	+	Dudaniec *et al.* 2006

Bearded Vulture	*Gypaetus barbatus*	ACC	Pull. vs. Ad.	+	Hernández and Margalida 2010
Herring Gull	*Larus argentatus*	CHA	Pull. vs. Ad.	+	Averbeck 1992
Masai Ostrich	*Struthio camelus**	STR	Juv. (1-12 months)	+	Samour *et al.* 2011
Mallard	*Anas platyrhynchos**	ANS	Juv. vs. Ad.	+	Oyewale *et al.* 1998
Emperor Penguin	*Aptenodytes forsteri*	SPH	Juv. vs. Ad.	(-)	Ponganis *et al.* 1999
Kori Bustard	*Ardeotis kori**	GRU	Juv. vs. Ad.	+	Howlett *et al.* 1998
Houbara	*Chlamydotis undulata macqueenii**	GRU	Juv. vs. Ad.	+	Howlett *et al.* 2002
Feral Pigeon	*Columba livia* f. *domestica*	COL	Juv. vs. Ad.	+	Pavlak *et al.* 2005
Rockhopper Penguin	*Eudyptes chrysocome*	SPH	Juv. vs. Ad.	+	Hawkey *et al.* 1989
Rufous-crested Bustard	*Eupodotis ruficrista gindiana**	GRU	Juv. vs. Ad.	+	Howlett *et al.* 2002
White-bellied Bustard	*Eupodotis senegalensis**	GRU	Juv. vs. Ad.	+	Howlett *et al.* 2002
Common Snipe	*Gallinago gallinago*	CHA	Juv. vs. Ad.	+	Minias *et al.* 2014
Hill Mynah	*Gracula religiosa**	PAS	Juv. vs. Ad.	(-)	Archawaranon 2005
Common Crane	*Grus grus*	GRU	Juv. vs. Ad.	(-)	Abelenda *et al.* 1993
Common Crane	*Grus grus*	GRU	Juv. vs. Ad.	(-)	Puerta *et al.* 1990
House Sparrow	*Passer domesticus*	PAS	Juv. vs. Ad.	(-)	Puerta *et al.* 1995
Common Pheasant	*Phasianus colchicus**	GAL	Juv. vs. Ad.	(-)	Keçeci and Çöl 2011
Chilean Flamingo	*Phoenicopterus chiliensis**	PHO	Juv. vs. Ad.	+	Hawkey *et al.* 1984a
American Flamingo	*Phoenicopterus ruber**	PHO	Juv. vs. Ad.	+	Hawkey *et al.* 1984b
Gentoo Penguin	*Pygoscelis papua*	SPH	Juv. vs. Ad.	+	Hawkey *et al.* 1989
Masai Ostrich	*Struthio camelus**	STR	Juv. vs. Ad.	+	Palomeque *et al.* 1991
Caperceillie	*Tetrao urogallus**	GAL	Juv. vs. Ad.	+	Lavin *et al.* 1992
Budgerigar	*Melopsittacus undulatus**	PSI	Ad. (1-6 years)	(-)	Harper and Lowe 1998
Common Snipe	*Gallinago gallinago*	CHA	Ad. (1-9 years)	(-)	Minias *et al.* 2014
Feral Pigeon	*Columba livia* f. *domestica*	COL	Ad. (1-17 years)	- (after 9th year)	Prinzinger and Misovic 2010
Black-browed Albatross	*Thalassarche melanophris*	PRO	Ad. (12-30 years)	(-)	Crossin *et al.* 2012

[A] ACC – Accipitriformes, ANS – Anseriformes, CIC – Ciconiiformes, CHA – Charadriiformes, COL – Columbiformes, COR – Coraciiformes, GAL – Galliformes, GRU – Gruiformes, PAS – Passeriformes, PHO – Phoenicopteriformes, PRO – Procellariformes, PSI – Psittaciformes, RHE – Rheiformes, SPH – Sphenisciformes, STR – Struthioniformes.

Table S2: Sex-related differences in hemoglobin concentrations in birds. Studies conducted in captivity are marked with an asterisk. Note: "(-)" indicates no differences in Hb concentration between sexes; F – females, M – males.

Common name	Specific name	Order[A]	Age	Hb concentration	Reference
White Stork	*Ciconia ciconia*	CIC	Pull.	F > M	Kaminski *et al.* 2014
Montagu's Harrier	*Circus pygargus*	ACC	Pull.	(-)	Limiñana *et al.* 2009
Parrot Finch	*Erythrura trichroa*	PAS	Pull.	F < M	Pryke and Rollins 2012
Great Cormorant	*Phalacrocorax carbo sinensis*	SUL	Pull.	(-)	Minias *et al.* 2013
Common Pheasant	*Phasianus colchicus**	GAL	Pull.	F > M	dos Santos Schmidt *et al.* 2007
Bearded Vulture	*Gypaetus barbatus*	ACC	Pull./Ad.	(-)	Hernández and Margalida 2010
Common Pheasant	*Phasianus colchicus**	GAL	Pull./Ad.	(-)	Keçeci and Çöl 2011
Lesser Rhea	*Pterocnemia pennata**	RHE	Pull./Ad.	(-)	Reissig *et al.* 2002
Mallard	*Anas platyrhynchos**	ANS	Ad.	(-)	Shave and Howard 1976
Mallard	*Anas platyrhynchos**	ANS	Ad.	(-)	Oyewale *et al.* 1998
Black Duck	*Anas superciliosa*	ANS	Ad.	(-)	Mulley 1979
Giant Canada Goose	*Branta canadensis maxima**	ANS	Ad.	(-)	Shave and Howard 1976
Cory's Shearwater	*Calonectris diomedea*	PRO	Ad.	(-)	Forero *et al.* 2006
Cory's Shearwater	*Calonectris diomedea*	PRO	Ad.	(-)	Navarro *et al.* 2007
Brown Skua	*Catharacta lonnbergii*	CHA	Ad.	(-)	Myrcha and Kostelecka-Myrcha 1980
Wood Duck	*Chemonetta jubata*	ANS	Ad.	(-)	Mulley 1980
Whiskered Tern	*Chlidonias hybrida*	CHA	Ad.	(-)	Minias 2014
Montagu's Harrier	*Circus pygargus*	ACC	Ad.	(-)	Lavin *et al.* 1993
Northern Bobwhite	*Colinus virginianus**	GAL	Ad.	(-)	Quinn *et al.* 2007
Feral Pigeon	*Columba livia* f. *domestica*	COL	Ad.	(-)	Kasprzak *et al.* 2006
Feral Pigeon	*Columba livia* f. *domestica*	COL	Ad.	F < M	Gayathri and Hegde 1994
Feral Pigeon	*Columba livia* f. *domestica*	COL	Ad.	(-)	Pavlak *et al.* 2005
Blue Tit	*Cyanistes caeruleus*	PAS	Ad.	F > M	Kaliński *et al.* 2012
Common Snipe	*Gallinago gallinago*	CHA	Ad.	(-)	Minias *et al.* 2014
Hill Mynah	*Gracula religiosa*	PAS	Ad.	(-)	Archawaranon 2005

Herring Gull	*Larus argentatus*	CHA	Ad.	(-)	Averbeck 1992
Kelp Gull	*Larus dominicus*	CHA	Ad.	(-)	Myrcha and Kostelecka-Myrcha 1980
Great Black-backed Gull	*Larus marinus*	CHA	Ad.	(-)	Averbeck 1992
Bar-tailed Godwit	*Limosa lapponica*	CHA	Ad.	(-)	Landys-Ciannelli *et al.* 2002
Bar-tailed Godwit	*Limosa lapponica*	CHA	Ad.	(-)	Piersma *et al.* 1996
Chestnut Mannikin	*Lonchura malacca*	PAS	Ad.	(-)	Mercurio *et al.* 2008
Crimson Finch	*Neochmia phaeton*	PAS	Ad.	F < M[B]	Milenkaya *et al.* 2013
Wilson's Storm Petrel	*Oceanites oceanicus*	PRO	Ad.	F > M	Myrcha and Kostelecka-Myrcha 1980
Great Tit	*Parus major*	PAS	Ad.	F > M	Kaliński *et al.* 2012
Common Pheasant	*Phasianus colchicus*	GAL	Ad.	(-)	dos Santos Schmidt *et al.* 2007
Ruff	*Philomachus pugnax*	CHA	Ad.	(-)	Banerjee and Banerjee 1977
Chilean Flamingo	*Phoenicopterus chilensis*	PHO	Ad.	(-)	Puerta *et al.* 1989
American Flamingo	*Phoenicopterus ruber**	PHO	Ad.	(-)	Hawkey *et al.* 1984b
Puna Ibis	*Plegadis ridgewayi**	CIC	Ad.	(-)	Coke *et al.* 2004
Roseringed Parakeet	*Psittaculla krameri**	PSI	Ad.	(-)	Nazifi and Vesal 2003
Adélie Penguin	*Pygoscelis adeliae*	SPH	Ad.	(-)	Myrcha and Kostelecka-Myrcha 1980
Chinstrap Penguin	*Pygoscelis antarctica*	SPH	Ad.	(-)	Myrcha and Kostelecka-Myrcha 1980
Gentoo Penguin	*Pygoscelis papua*	SPH	Ad.	(-)	Myrcha and Kostelecka-Myrcha 1980
Antarctic Tern	*Sterna vittata*	CHA	Ad.	(-)	Myrcha and Kostelecka-Myrcha 1980
Kakapo	*Strigops habroptilus*	PSI	Ad.	(-)	Low *et al.* 2006
White Ibis	*Threskiornis melanocephalus**	CIC	Ad.	(-)	Aengwanich and Tanomtong 2004

[A] ACC – Accipitriformes, ANS – Anseriformes, CIC – Ciconiiformes, CHA – Charadriiformes, COL – Columbiformes, GAL – Galliformes, PAS – Passeriformes, PHO – Phoenicopteriformes, PRO – Procellariformes, PSI – Psittaciformes, RHE – Rheiformes, SPH – Sphenisciformes, Sul – Suliformes.

[B] only during egg laying and incubation

References:

Abelenda M, Nava MP, Fernandez A, Alonso JA, Alonso JC, Muñoz-Pulido R, Bautista LM, Puerta ML (1993) Blood values of common cranes (*Grus grus*) by age and season. *Comp Biochem Physiol A* 104: 575–578.

Aengwanich W, Tanomtong A (2004) Hematological and serum biochemical values of white ibis (*Threskiornis melanocephalus*). *Songklanakarin J Sci Technol* 26: 823–828.

Archawaranon M (2005) Hematological investigations of captive Hill Mynah *Gracula religiosa* in Thailand. *Int J Poultry Sci* 4: 679–682.

Arnold G, Lill A, Baldwin J (1999) Development of some aspects of blood oxygen transport in nestling short-tailed shearwaters. *Aust J Zool* 47: 479–487.

Averbeck C (1992) Haematology and blood chemistry of healthy and clinically abnormal great black-backed gulls (*Larus marinus*) and herring gulls (*Larus argentatus*). *Avian Pathol* 21: 215–223.

Banerjee V, Banerjee M (1977) Variations of erythrocyte numbers and haemoglobin content of a migratory bird: *Philomachus pugnax* (Linnaeus). *Zool Anz Jena* 199: 261–264.

Bech C, Klaassen M (1996) Blood hemoglobin content and metabolic performance of Arctic Tern chicks *Sterna paradisaea*. *J Avian Biol* 27: 112–117.

Bolton AJ, Lill A, Baldwin J (1999) Haematological changes during development of the noisy miner: implications for oxygen transport. *Aust J Zool* 47: 455–461.

Coke RL, West GD, Hoover JP (2004) Hematology and plasma biochemistry of captive puna ibis (*Plegadis ridgewayi*). *J Wildl Dis* 40: 141–144.

Crossin GT, Phillips RA, Trathan PN, Fox DS, Dawson A, Wynne-Edwards KE, Williams TD (2012) Migratory carryover effects and endocrinological correlates of reproductive decisions and reproductive success in female albatrosses. *Gen Comp Endocr* 176: 151–157.

Deaton KE, Bishop CM, Butler PJ (1998) Tissue-specific effects of hypothyroidism on postnatal muscle development in the barnacle goose. *J Exp Biol* 201: 827–836.

dos Santos Schmidt EM, Paulillo AC, Santin E, Dittrich RL, de Oliveira EG (2007) Hematological and serum chemistry values for the ring-necked pheasant (*Phasianus colchicus*): variation with sex and age. *Int J Poultry Sci* 6: 137–139.

Dudaniec RY, Kleindorfer S, Fessl B (2006) Effects of the introduced ectoparasite *Philornis downsi* on haemoglobin level and nestling survival in Darwin's small ground finch (*Geospiza fuliginosa*). *Austral Ecol* 31: 88–94.

Eklom K, Lill A (2006a) Development pattern of blood oxygen carrying capacity in rainbow bee-eater nestlings. *Aust J Zool* 54: 1–7.

Eklom K, Lill A (2006b) Development of parameters influencing blood oxygen-carrying capacity in nestling doves. *Emu* 106: 283–288.

Forero MG, González-Solis J, Igual JM, Hobson KA, Ruíz X, Viscor G (2006) Ecological and physiological variance in T-cell mediated immune response in Cory's shearwaters. *Condor* 108: 865–876.

Gayathri KL, Hegde SN (1994) Sexual differences in blood values of the pigeon, *Columba livia*. *Comp Biochem Physiol B* 109: 219–224.

Gayathri KL, Shenoy KB, Hegde SN (2004) Blood profile of pigeons (*Columba livia*) during growth and breeding. *Comp. Biochem. Physiol. A* 138: 187–192.

Haggblom L, Terwilliger RC, Terwilliger NB (1988) Changes in myoglobin and lactate dehydrogenase in muscle tissues of a diving bird, the pigeon guillemot, during maturation. *Comp Biochem Physiol B* 91: 273–277.

Harper EJ, Lowe B (1998) Hematology values in a colony of budgerigars (*Melopsittacus undulatus*) and changes associated with aging. *J Nutr* 128: 2639S–2640S.

Hawkey C, Horsley DT, Keymer IF (1989) Haematology of wild penguins (Spenisciformes) in the Falkland Islands. *Avian Pathol* 18: 495–502.

Hawkey C, Hart MG, Samour HJ (1984) Age-related haematological changes and haemopathological responses in Chilean flamingos (*Phoenicopterus chiliensis*). *Avian Pathol* 13: 223–229.

Hawkey C, Hart MG, Samour HJ, Knight JA, Hutton RE (1984) Haematological findings in healthy and sick captive rosy flamingos (*Phoenicopterus ruber ruber*). *Avian Pathol* 13: 163–172.

Hernández M, Margalida A (2010) Hematology and blood chemistry reference values and age-related changes in wild bearded vultures (*Gypaetus barbatus*). *J Wildl Dis* 46: 390–400.

Howlett JC, Bailey TA, Samour JH, Naldo JL, D'Aloia MA (2002) Age-related hematologic changes in captive-reared houbara, white-bellied, and rufous-crested bustards. *J Wildl Dis* 38: 804–816.

Howlett JC, Samour JH, Bailey TA, Naldo JL (1998) Age-related haematology changes in captive-reared kori bustards (*Ardeotis kori*). *Comp Haematol Int* 8: 26–30.

Kaliński A, Bańbura M, Skwarska J, Wawrzyniak J, Zieliński P, Glądalski M, Markowski M, Bańbura J (2012) Parallel variation in haemoglobin concentration in nestling-rearing Blue Tits *Cyanistes caeruleus* and Great Tits *Parus major*. *Acta Ornithol* 47: 129–136.

Kaminski P, Jerzak L, Sparks TH, Johnston A, Bochenski M, Kasprzak M, Wiśniewska E, Mroczkowski S, Tryjanowski P (2014) Sex and other sources of variation in the haematological parameters of White Stork *Ciconia ciconia* chicks. *J Ornithol* 155: 307–314.

Kasprzak M, Hetmański T, Kulczykowska E (2006) Changes in hematological parameters in free-living pigeons (*Columba livia* f. *urbana*) during the breeding cycle. *J Ornithol* 147: 599–604.

Kececi T, Col R (2011) Haematological and biochemical values of the blood of pheasants (*Phasianus colchicus*) of different ages. *Turk J Vet Anim Sci* 35: 149–156.

Kostelecka-Myrcha A, Jaroszewicz M (1993) The changes in the values of red blood indices during the nestling development of the House Martin *Delichon urbica*. *Acta Ornithol* 28: 39–46.

Kostelecka-Myrcha A, Myrcha A (1989) Changes of the red blood picture during nestling development of Wilson's storm petrel (*Oceanites oceanicus* Kuhl). *Pol Polar Res* 10: 151–162.

Kostelecka-Myrcha A, Pinowski J, Tomek T (1970) Changes in respiratory function of the blood of nestling Tree Sparrows (*Passer m. montanus* L.) during their development. *Bull Acad Pol Sci* 18: 717–722.

Kostelecka-Myrcha A, Pinowski J, Tomek T (1971) Changes of the respiratory function of blood of nestling common sparrows *Passer domesticus* L. during their development. *Bull Acad Pol Sci* 19: 639–645.

Kostelecka-Myrcha A, Pinowski J, Tomek T (1972) Changes in respiratory function of the blood of nestling starlings (*Sturnus v. vulgaris* L.) during their development. *Bull Acad Pol Sci* 20: 373–378.

Kostelecka-Myrcha A, Pinowski J, Tomek T (1973) Changes in the hematological values during the nestling period of the Great Tit (*Parus major* L.). *Bull Acad Pol Sci* 21: 725–732.

Kostelecka-Myrcha A, Zukowski J, Oksiejczuk E (1996) Changes in the red blood indices during nestling development of the Tree Sparrow *Passer montanus* in an urban environment. *Ibis* 139: 92–96.

Landys-Ciannelli MM, Jukema J, Piersma T (2002) Blood parameter changes during stopover in a long-distance migratory shorebird, the bar-tailed godwit *Limosa lapponica taymyrensis*. *J Avian Biol* 33: 451–455.

Lavin S, Cuenca R, Marco I, Pastor J, Vinas L (1993) Hematological values of montagu's harrier (*Circus pygargus*). *Comp Biochem Physiol A* 105: 103–104.

Lavin S, Cuenca R, Marco I, Velarde R, Vinas L (1992) Haematology and blood biochemistry of capercaillie (*Tetrao urogallus*). *Avian Pathol* 21: 711–715.

Limiñana R, López-Olvera JR, Gallardo M, Fordham M, Urios V (2009) Blood chemistry and hematologic values in free-living nestlings of Montagu's Harriers (*Circus pygargus*) in a natural habitat. *J Zoo Wildl Med* 40: 687–695.

Low M, Eason D, Elliott G, McInnes K, Paul-Murphy J (2006) Hematologic and biochemical reference ranges for the kakapo (*Strigops habroptilus*): generation and interpretation in a field-based wildlife recovery program. *J Avian Med Surg* 20: 80–88.

Mercurio DG, Marte BRG, Cruzana BC (2009) Hematological values of chestnut mannikin (*Lonchura malacca*) caught in Laguna. *Philipp J Vet Med* 45: 63–66.

Milenkaya O, Weinstein N, Legge S, Walters JR (2013) Variation in body condition indices of crimson finches by sex, breeding stage, age, time of day, and year. *Conserv Physiol* 1, doi: 10.1093/conphys/cot020.

Minias P (2014) High glucose concentrations are associated with symptoms of mild anaemia in Whiskered Terns: consequences for assessing physiological quality in birds. *J Ornithol* 155: 1067–1070.

Minias P, Kaczmarek K, Janiszewski T, Markowski J (2013) Hematology and plasma biochemistry values of Great Cormorant (*Phalacrocorax carbo sinensis*) nestlings. *J Wildl Dis* 49: 194–196.

Minias P, Włodarczyk R, Piasecka A, Kaczmarek K, Janiszewski T (2014) Ecological, physiological and morphological correlates of blood hemoglobin concentration in a migratory shorebird. *Physiol Biochem Zool* 87: 771–781.

Montesinos A, Sainz A, Pablos MV, Mazzucchelli F, Tesouro MA (1997) Hematological and plasma biochemical reference intervals in young white storks*. J Wildl Dis* 33: 405–412.

Mulley RC (1979) Haematology and blood chemistry of the black duck *Anas superciliosa*. *J Wildl Dis* 15: 437–441.

Mulley RC (1980) Haematology of the wood duck, *Chenonetta jubata*. *J Wildl Dis* 16: 271–273.

Myrcha A, Kostelecka-Myrcha A (1980) Hematological studies on Antarctic birds. I. Hematological indices in some species of the birds studied during Australian summer. *Pol Polar Res* 1: 169−173.

Navarro J, González-Solís J, Viscor G (2007) Nutritional and feeding ecology in Cory's shearwater *Calonectris diomedea* during breeding. *Mar Ecol Prog Ser* 351: 261−271.

Nazifi S, Vesal N (2003) Hematological values of healthy roseringed parakeets (*Psittaculla krameri*). *J Appl Anim Res* 24: 165−168.

O'Dwyer TW, Buttemer WA, Priddel DM (2007) Differential rates of offspring provisioning in Gould's petrels: are better feeders better breeders. *Aust J Zool* 55: 155−160.

Oyewale JO, Olayemi FO, Rahman SA (1998) Blood characteristics of the Nigerian local duck (*Anas platyrhynchos*). I. Red blood cell characteristics. *Vet Arh* 68: 199–204.

Palomeque J, Pinto D, Viscor G (1991) Hematologic and blood chemistry values of the Masai ostrich (*Struthio camelus*). *J Wildl Dis* 27: 34–40.

Pavlak M, Vlahović K, Jerčić J, Dovč A, Župančić Ž (2005) Age, sexual and seasonal differences of haematological values and antibody status to *Chlamydophila* sp. in feral and racing pigeons (*Columba livia* forma *domestica*) from an urban environment (Zagreb, Croatia). *Eur J Wildl Res* 51: 271–276.

Piersma T, Everaarts JM, Jukema J (1996) Build-up of red blood cells in refuelling bar-tailed godwits in relation to individual migratory quality. *Condor* 98: 363−370.

Ponganis PJ, Starke LN, Horning M, Kooyman GL (1999) Development of diving capacity in Emperor Penguins. *J Exp Biol* 202: 781−786.

Prinzinger R, Misovic A (2010) Age-correlation of blood values in the rock pigeon (*Columba livia*). *Comp Biochem Physiol A* 156: 351–356.

Pryke SR, Rollins LA (2012) Mothers adjust offspring sex to match the quality of the rearing environment. *Proc R Soc B* 279: 4051−4057.

Puerta ML, Alonso JC, Huecas V, Alonso JA, Abelenda M, Munoz-Pulido R (1990) Hematology and blood chemistry of wintering Common Cranes. *Condor* 92: 210–214.

Puerta ML, Campo AL, Abelenda M, Fernandez A, Huecas V, Nava MP (1992) Hematological trends in flamingos, *Phoenicopterus ruber*. *Comp Biochem Physiol A* 102: 683−686.

Puerta ML, Huecas V, Del Campo AL (1989) Hematology and blood chemistry of the Chilean flamingo. *Comp Biochem Physiol A* 94: 623−625.

Puerta M, Nava MP, Venero C, Veiga JP (1995) Hematology and plasma chemistry of house sparrows (*Passer domesticus*) along the summer months and after testosterone treatment. *Comp Biochem Physiol A* 110: 303−307.

Quinn MJ, Bazar MA, McFarland CA, Perkins EJ, Gust KA, Gogal RM, Johnson MS (2007) Effects of subchronic exposure to 2, 6-dinitrotoluene in the northern bobwhite (*Colinus virginianus*). *Environ Toxicol Chem* 26: 2202−2207.

Reissig EC, Robles CA, Sager R (2002) Hematology and serum chemistry values of the lesser rhea (*Pterocnemia pennata*) raised in Patagonian farms (Argentina). *J Zoo Wildl Med* 33: 328−331.

Samour J, Naldo J, Libanan N, Rahman H, Sakkir M (2011) Age-related hematology and plasma chemistry changes in captive masai ostriches (*Struthio camelus massaicus*). *Comp Clin Pathol* 20: 659−667.

Shave HJ, Howard V (1976) A hematologic survey of captive waterfowl. *J Wildl Dis* 12: 195–201.

Simmons P, Lill A (2006) Development of parameters influencing blood oxygen carrying capacity in the welcome swallow and fairy martin. *Comp Biochem Physiol A* 143: 459−468.

Whitworth TL, Bennett GF (1992) Pathogenicity of larval *Protocalliphora* (Diptera: Calliphoridae) parasitizing nestling birds. *Can J Zool* 70: 2184−2191.
